# Supplementary material for: How informative were early SARS-CoV-2 treatment and prevention trials? a longitudinal cohort analysis of trials registered on ClinicalTrials.gov
Source: PLoS One. 2022 Jan 21;17(1):e0262114. doi: 10.1371/journal.pone.0262114 (PMC8782516; doi:10.1371/journal.pone.0262114)
Supplement: S2 Fig — (DOCX) [file pone.0262114.s003.docx]

**S2 Figure. Ratio of Actual to Estimated Number of Patients Enrolled**

Vertical red line indicates 85% goal enrollment (threshold for recruitment feasibility)
