# Supplementary material for: How informative were early SARS-CoV-2 treatment and prevention trials? a longitudinal cohort analysis of trials registered on ClinicalTrials.gov
Source: PLoS One. 2022 Jan 21;17(1):e0262114. doi: 10.1371/journal.pone.0262114 (PMC8782516; doi:10.1371/journal.pone.0262114)
Supplement: S1 Table — (DOCX) [file pone.0262114.s005.docx]

**S1 Table. Inter-rater Agreement**

| **Category** | **Unweighted Cohen’s Kappa** |
| --- | --- |
| Screening of trials for inclusion in the cohort | 0.844 |
| Distinguishing treatment and prevention trials | 0.799 |
| Evaluation of location of care | 0.663 |
| Evaluation of COVID-19 disease severity | 0.516 |
| Distinguishing presence versus absence of comparator arm | 0.889 |
| Categorization of placebo versus standard of care control arm | 0.834 |
| Evaluation of reason for termination | 0.921 |
| Assessment of redundancy | 0.452 |
