# Supplementary material for: How informative were early SARS-CoV-2 treatment and prevention trials? a longitudinal cohort analysis of trials registered on ClinicalTrials.gov
Source: PLoS One. 2022 Jan 21;17(1):e0262114. doi: 10.1371/journal.pone.0262114 (PMC8782516; doi:10.1371/journal.pone.0262114)
Supplement: S2 Table — (DOCX) [file pone.0262114.s006.docx]

**S2 Table. Additional Characteristics of Trial Cohort**

| **Category** | **Number of Trials**  **(N = 500)** |
| --- | --- |
| Age of Trial Participants |  |
| Includes pediatric population^a^ | 49 |
| Includes elderly population^b^ | 493 |
| Location of Care^c^ |  |
| Ambulatory | 130 |
| Hospitalized | 368 |
| Intensive Care | 150 |
| Location of Care Not Specified | 19 |
| SARS-CoV-2 Severity^d^ |  |
| Healthy (no SARS-CoV-2 infection) | 77 |
| Asymptomatic | 51 |
| Mild | 110 |
| Moderate | 228 |
| Severe | 251 |
| Critical | 150 |
| Severity Not Specified | 26 |

a. Includes participants < 18 years of age.

b. Includes participants > 59 years of age.

c. Trials may include patients from more than one location of care

d. Trials may include patients from more than one severity category
