# Supplementary material for: How informative were early SARS-CoV-2 treatment and prevention trials? a longitudinal cohort analysis of trials registered on ClinicalTrials.gov
Source: PLoS One. 2022 Jan 21;17(1):e0262114. doi: 10.1371/journal.pone.0262114 (PMC8782516; doi:10.1371/journal.pone.0262114)
Supplement: S3 Table — (DOCX) [file pone.0262114.s007.docx]

**S3 Table. Range of Anticipated and Actual Enrollment**

| **Category** | **Number of Trials**  **(N = 500)** | **Median (Range) Anticipated Enrollment^a^** | **Median (Range) Actual Enrollment^b^** |
| --- | --- | --- | --- |
| Trial Phase |  |  |  |
| Phase 1/2 & Phase 2 | 290 | 100 (5-10000) | 60 (1-3217) |
| Phase 2/3 & Phase 3 | 210 | 400 (10-15000) | 241 (0-4891) |
| Randomization |  |  |  |
| Randomized | 423 | 200 (15-15000) | 142 (0-4891) |
| Non-Randomized | 30 | 73 (10-2944) | 38 (1-325) |
| NA^c^ | 47 | 37 (5-10000) | 27 (1-3217) |
| Trial Status^d^ |  |  |  |
| Completed | 54 | 100 (5-5000) | 100 (5-4891) |
| Terminated | 16 | 265 (40-1660) | 62 (1-299) |
| Active, Not Recruiting | 71 | 240 (5-10000) | 177 (1-3217) |
| Recruiting | 335 | 152 (9-15000) | 143 (0-4506) |
| Enrolling by Invitation | 11 | 128 (20-988) | 72 (30-231) |
| Suspended | 13 | 308 (30-900) | 27 (1-122) |
| Trial Type |  |  |  |
| Treatment Trial | 424 | 130 (5-12000) | 100 (0-4891) |
| Prevention Trial | 66 | 672 (20-15000) | 554 (1-4257) |
| Treatment & Prevention | 10 | 782 (164-3040) | 741 (143-2300) |
| Sponsorship |  |  |  |
| Industry Sponsor | 112 | 195 (20-8640) | 187 (16-4891) |
| Non-Industry Sponsor | 388 | 177 (5-15000) | 100 (0-4506) |
| Country Location |  |  |  |
| USA Trial | 179 | 200 (10-15000) | 95 (1-4891) |
| Non-USA Trial | 321 | 165 (5-12000) | 121 (0-4257) |
| Number of Centers |  |  |  |
| Single Center | 198 | 100 (5-10000) | 60 (1-4257) |
| Multicenter | 302 | 226 (10-15000) | 143 (0-4891) |
