# Supplementary material for: How informative were early SARS-CoV-2 treatment and prevention trials? a longitudinal cohort analysis of trials registered on ClinicalTrials.gov
Source: PLoS One. 2022 Jan 21;17(1):e0262114. doi: 10.1371/journal.pone.0262114 (PMC8782516; doi:10.1371/journal.pone.0262114)
Supplement: S1 File — (DOCX) [file pone.0262114.s008.docx]

**S1 File. Trial Inclusion and Exclusion Criteria**

Inclusion criteria:

- Interventional clinical trials
- Trial Status: Completed, Terminated, Suspended, Active not recruiting, Enrolling by invitation, Recruiting
- Trials testing an efficacy hypothesis in the primary outcome
- Phase numbers: Phase 1/2, Phase 2, Phase 2/3, Phase 3
- Drug, biological, surgical, radiotherapy, procedure and device interventions

Exclusion criteria:

- Behavioral interventions (including psychological, dietary, physiotherapy, etc.)
- Trials of natural products (not regulated by the FDA)
- Phase 1 trials (do not typically evaluate efficacy outcomes and are not required by FDA to register)
