# Supplementary material for: How informative were early SARS-CoV-2 treatment and prevention trials? a longitudinal cohort analysis of trials registered on ClinicalTrials.gov
Source: PLoS One. 2022 Jan 21;17(1):e0262114. doi: 10.1371/journal.pone.0262114 (PMC8782516; doi:10.1371/journal.pone.0262114)
Supplement: S2 File — (DOCX) [file pone.0262114.s009.docx]

**S2 File. Clinicaltrials.gov Search Criteria**

We downloaded clinical trial data directly as a zipped folder of XML files from the web front-end of ClinicalTrials.gov.

We used the following search criteria:

411 records identified through 12/01/2021:

Condition or disease: “Covid-19”

Study Type: “Interventional Studies”

Trial Status: “Recruiting, “Active, not recruiting,” “Completed,” “Enrolling by invitation,” “Suspended,” “Terminated”

Phase: Phase 2, Phase 3

Start Date: 01/01/2020 to 05/31/2020

110 records identified through 01/04/2021:

Condition or disease: “Covid-19”

Study Type: “Interventional Studies”

Trial Status: “Recruiting, “Active, not recruiting,” “Completed,” “Enrolling by invitation,” “Suspended,” “Terminated”

Phase: Phase 2, Phase 3

Start Date: 06/01/2020 to 06/30/2020
