# Supplementary material for: How informative were early SARS-CoV-2 treatment and prevention trials? a longitudinal cohort analysis of trials registered on ClinicalTrials.gov
Source: PLoS One. 2022 Jan 21;17(1):e0262114. doi: 10.1371/journal.pone.0262114 (PMC8782516; doi:10.1371/journal.pone.0262114)
Supplement: S3 File — (DOCX) [file pone.0262114.s010.docx]

**S3 File. Data Downloaded from ClinicalTrials.gov**

- NCT Number
- Link to ClinicalTrials.gov record
- Title of Trial
- Indication
- Intervention
- Phase
- Enrollment (current actual or anticipated enrollment and original estimated enrollment)
- Age
- Gender
- Trial status
- Reason for termination/suspension (for trials that are terminated or suspended)
- Allocation (randomization)
- Interventional model (Single group versus multiple group assignment)
- Masking
- Trial Start date (current start date and original start date)
- Trial projected close date
- Lead sponsor
- Funder type
- Number of arms
- Study location (country location of each center participating in the trial)
- Primary outcome
