# Supplementary material for: How informative were early SARS-CoV-2 treatment and prevention trials? a longitudinal cohort analysis of trials registered on ClinicalTrials.gov
Source: PLoS One. 2022 Jan 21;17(1):e0262114. doi: 10.1371/journal.pone.0262114 (PMC8782516; doi:10.1371/journal.pone.0262114)
Supplement: S4 File — (DOCX) [file pone.0262114.s011.docx]

**S4 File. Additional Data Points**

Data points requiring human curation were independently extracted by two individuals using Numbat software – an open source tool created by BGC, available at <https://numbat.bgcarlisle.com>.

This included the following items:

- Categorization of trial type (SARS-CoV-2 Treatment trial versus Prevention trial versus unclear/not stated).
  - A SARS-CoV-2 treatment trial was defined as a clinical trial conducted in a population with documented SARS-CoV-2 infection designed to evaluate the safety and/or efficacy of an intervention directed at improving clinical or surrogate outcomes related to the disease.
  - A SARS-CoV-2 prevention trial was defined as a clinical trial conducted in a population without known SARS-CoV-2 infection designed to evaluate the safety and/or efficacy of an intervention directed at preventing serologic conversion and/or clinical disease due to SARS-CoV-2. These studies are often conducted in an at-risk population, whereby exposure to SARS-CoV-2 is felt to be likely. They can include populations who have been exposed to SARS-CoV-2 (post-exposure prophylaxis), without documented infection or clinical symptoms of disease, in addition to populations who may be exposed to SARS-CoV-2 at a future time (pre-exposure prophylaxis).
- Location of care (Ambulatory versus Hospitalized versus Intensive Care versus unclear/not stated)
- Illness severity (Healthy patient versus Asymptomatic versus Mild versus Moderate versus Severe versus Critical versus unclear/not stated)
- Illness severity was as stated by investigators, or, if this was not provided, it was inferred using the WHO COVID-19 disease severity classification.^1^
- Treatment type (guided by the World Health Organization (WHO) COVID Classification of treatment types^2^
- Presence of a comparator trial arm
- Presence of a placebo or standard of care trial arm
- Primary outcome classification (Clinical outcome versus Surrogate outcome versus Procedural outcome versus Test result (the latter was designated for prevention trials)
- A clinical outcome is one that specifically evaluate quality of life, morbidity/disability or mortality. Examples of COVID-19 clinical outcomes from^3^: i) all-cause mortality; ii) respiratory failure (including need for mechanical ventilation, ECMO, non-invasive ventilation, high-flow nasal cannula); iii) need for intensive care unit level care; iv) need for hospitalization; v) objective measures of sustained improvement (e.g. return to baseline oxygen requirement); vi) sustained clinical recovery. Clinical outcomes may be presented on their own (as a single clinical outcome), as a composite outcome, or as an ordinal scale combining multiple clinical outcomes.
- A surrogate outcome is one that does not directly measure the impact of an intervention on quality of life, morbidity/disability or mortality. Rather, it is designed to predict a clinical outcome. For example, virologic response (virologic clearance / viral load) is an example of a COVID-19 surrogate.
- Inclusion of elderly patients (aged 60 years and above)
- For terminated trials, classification of reason for termination (based on that stated in the “why stopped?” field in the trial registration record (Feasibility versus Efficacy/Safety/Progress of Science versus unclear/not stated)

**References**

1. World Health Organization. Clinical management of COVID-19 Interim Guidance. 27 May 2020. <https://apps.who.int/iris/bitstream/handle/10665/332196/WHO-2019-nCoV-clinical-2020.5-eng.pdf>. Accessed 2020-09-24.

2. World Health Organization. WHO R&D Blueprint COVID 19 Experimental Treatments. 2020.

3. U.S. Food & Drug Association. COVID 19: Developing Drugs and Biological Products for Treatment or Prevention, Guidance for Industry. May 2020. <https://www.fda.gov/media/137926/download> Accessed June 18 2020.
