# Supplementary material for: How informative were early SARS-CoV-2 treatment and prevention trials? a longitudinal cohort analysis of trials registered on ClinicalTrials.gov
Source: PLoS One. 2022 Jan 21;17(1):e0262114. doi: 10.1371/journal.pone.0262114 (PMC8782516; doi:10.1371/journal.pone.0262114)
Supplement: S5 File — (DOCX) [file pone.0262114.s012.docx]

**S5 File. Assessment of Trial Redundancy**

Assessment of trial redundancy included both automated and human-curated steps. Using datapoints directly downloaded from ClinicalTrials.gov and those previously independently assessed by KK & LZ, an automated comparison of trial phase, type of trial (treatment versus prevention), location of care, illness severity, age (binary assessment of inclusion/exclusion of population aged ≥ 60), and treatment regimen (using human-curated synonyms for interventions) was carried out using R version 3.6.3. Trials with a direct match on all the above elements were advanced to independent assessment of trial primary outcome, comparator arms and other (including type of population (for example, healthcare workers) and trial aim (for example, pre-exposure prophylaxis versus post-exposure prophylaxis) by two authors (NH & KK).

Trials were classified as redundant if there was a comparator trial with an earlier start date of the same phase, location of care, severity, age, treatment regimen, primary outcome (primary outcome of the earlier comparator trial had to at least encompass that of the trial of interest), treatment arms (arms of the earlier comparator trial had to at least match those in the trial of interest), population and aim.
