# Supplementary material for: How informative were early SARS-CoV-2 treatment and prevention trials? a longitudinal cohort analysis of trials registered on ClinicalTrials.gov
Source: PLoS One. 2022 Jan 21;17(1):e0262114. doi: 10.1371/journal.pone.0262114 (PMC8782516; doi:10.1371/journal.pone.0262114)
Supplement: S6 File — (DOCX) [file pone.0262114.s013.docx]

**S6 File. Protocol Deviations**

1. We redefined the terms for our measures of effect as follows:

- “Importance” as “Potential redundancy”,
- “Feasibility” as “Feasibility of Patient-Participant Recruitment”.

2. We calculated the proportion of trials meeting all 3 criteria of informativeness.

3. We performed a *post hoc* assessment of trial similarity, based on assessment of trial type, phase, patient-participant characteristics and treatment regimen.

4. We will be publishing the results of our analysis of feasibility of recruitment by location in a separate manuscript.

5. Based on reviewer recommendations, for our stratified analysis (in Table 3) we calculated the difference between proportions and presented a 95% confidence interval for that difference.
